# Applications of gradient index metamaterials in waveguides

**DOI:** 10.1038/srep18223

**Published:** 2015-12-14

**Authors:** Yangyang Fu, Yadong Xu, Huanyang Chen

**Affiliations:** 1 College of Physics, Optoelectronics and Energy & Collaborative Innovation Center of Suzhou Nano Science and Technology, Soochow University, No.1 Shizi Street, Suzhou 215006, China

## Abstract

In this letter, we find that gradient index metamaterials (GIMs) could be utilized to manipulate wave propagation in waveguides. Through manipulating the conversion between propagating wave and surface wave, we can design some interesting applications in waveguides, such as controlling transmission effect, realizing bending waveguide and achieving waveguide splitting effect. These devices not only work for both transverse electric and magnetic polarized waves, but also function for a broadband of spectra. Numerical simulations are performed to verify our findings.

At all times, manipulating propagation of electromagnetic (EM) wave is a very significant topic in optic community. In particular, in recent years, such topic has made remarkable progress due to the rapid development of transformation optics (TO)^1^,^2^,^3^ and metamaterials^4^,^5^. On basis of TO, one can in principle design the propagating path for light at will. But the required materials almost are anisotropic and inhomogeneous, which is not attainable in nature. This fatal weakness restricts the further development of TO until the advent of metamaterials, which have been employed to experimentally realize the first invisibility cloak^6^ designed by TO. After that, metamaterials have drawn extensive attention and more applications are proposed such as electromagnetic concentrators^7^,^8^, field rotators^9^,^10^ and so forth. More recently, as a special kind of metamaterials, zero index metamaterials (ZIMs) are also used to manipulate the propagation of light, *e.g.,* realizing bending waveguide^11^,^12^, controlling total transmission or reflection^13^,^14^,^15^,^16^ in a waveguide, achieving asymmetric transmission^17^ and inducing inhomogeneous field via cavity modes^18^. Moreover, controlling the propagation of light is also realized by metasurfaces^19^,^20^,^21^, which could be regarded to change the conventional laws of reflection and refraction. By utilizing the metasurfaces concept, when the gradient index metamaterials (GIMs) with a sub-wavelength thickness are placed in a waveguide, the asymmetric propagations^22^ of light can be obtained. In fact, such propagation characteristic is caused by mode conversion. In this work, based on the concept of mode conversion, we design a new kind of waveguides with GIMs. By manipulating the conversion between propagating wave and surface wave in waveguides, some interesting applications could be proposed, such as tunable transmission effect, waveguide bending and splitting effect. Numerically, we find that such devices can function for a broad band of spectra and are not restricted to polarizations of light. The design of mode convertor^23^ can also be realized by using the technique of TO, yet with more complicated material parameters and narrow working spectra.

## Results

### Model and Theory

**Figure 1:**
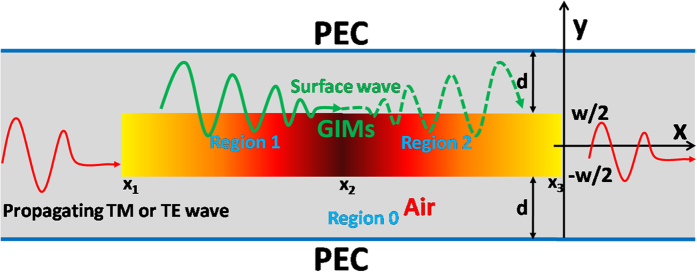
The schematic diagram of a straight waveguide structure with GIMs. Region 1 and region 2 are composed of GIMs with a width of *w*, and located at the center of an empty waveguide. The two parallel blue lines are PEC walls. The distance between GIMs and PEC walls are both *d*.

Let us start from a schematic diagram with the proposed new kind of waveguide structure with GIMs, as shown in Fig. 1. Region 1 and region 2 are made of GIMs with a width of *w*, which is located at the center of the waveguide. Region 0 marked by gray is air, and the air gaps between GIMs and the outer boundaries of the waveguide (now are perfect electrical conductor walls, or PEC walls) are both *d* . The effective permittivity of GIMs in region 1 is a function of position, which can be written as,


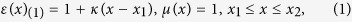


where 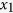 is the starting point of GIMs in region 1, 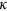 is a gradient ratio. While for the effective permittivity of GIMs in region 2, it is expressed as,


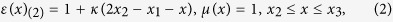


where 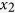 is the starting point of GIMs in region 2, and 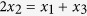. Based on Eq.(1) and(2), for the GIMs in region 1, the refractive index increases with the position varying from 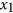 to 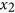, while for region 2, the refractive index decreases with the position varying from 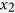 to 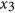. It is noted that, for a waveguide structure with GIMs, it is difficult to obtain its band structures. However, by replacing the GIMs with a kind of dielectric material which has the same refractive index as that of GIMs at a particular position, the dispersion relationship can be figured out, which has been investigated in Ref. 22 in detail. Through analyzing the band structures for a given working frequency, when the refractive index of GIMs varies from a low value into a higher one, the incident propagating wave will be gradually converted into an evanescent wave confined in the layer of GIMs, called as surface wave here. On the strength of such waveguide mode conversion^22^, the asymmetric propagation of light could be achieved. Sharing the concept of mode conversion in Ref. 22by designing a new kind of waveguides with GIMs as shown in Fig. 1, it is possible for us to manipulate EM wave in a new way. In order to obtain the feature of mode conversion, we analyze the band structures of our new waveguide with GIMs. As we follow the identical theory in Ref. 22here we will directly give out the corresponding designed results. For example, we set the width of the waveguide as *t* = 30 *mm*, that is 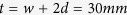, and the width of the GIMs is 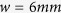. For such a waveguide structure, there is a cutoff frequency (currently 5 GHz) for transverse electric (TE) modes (electric field along *z* direction), while for transverse magnetic (TM) modes (magnetic field along *z* direction), there is no cutoff frequency. As we know, the dielectric materials are almost dispersionless and lossless in microwave frequencies. By means of drilling holes on the dielectric materials^24^,^25^ with different radii, and using the effective medium theory, the GIMs could be effectively manufactured. In addition, another structure such as a high-index dielectric strip with a gradient thickness (*e.g.*, zero thickness at 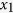 and 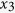, and a maximal value at 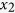), can realize similar functionality of the above GIMs and is much easier in fabrications (see in Supplementary Figure 1). We set the working frequency as 9.5 GHz. For simplicity, we consider the first mode for TE modes, *i.e.,* TE_1_ mode, or the zero-*th* mode for TM modes, *i.e.,* TM_0_ mode. When the incident wave (TE_1_ mode or TM_0_ mode) propagates at the GIMs in region 1, with the refractive index of GIMs increasing, the propagating wave will be gradually converted into surface wave. While for the surface wave in region 2, with the refractive index of GIMs decreasing, the surface wave will be gradually converted into propagating wave. Such a process in the waveguide with GIMs is visualized by the green curves in Fig. 1. We will take advantage of such a propagating property to design some meaningful applications in waveguides.

### Manipulating transmission effect

**Figure 2:**
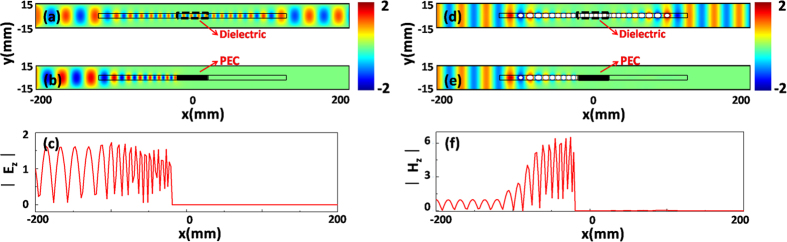
The simulated filed patterns and distributions for the straight waveguide structure. (**a**,**b**) are the corresponding electric field patterns for the cases of inserting the dielectric material and PEC into the middle of GIMs, respectively. **(c)** is the electric field distribution from *x* = −200 *mm* to *x* = 200 *mm* (y = 0 *mm*) in (**b**). (**a**), (**b**,**c**) are the cases for the incident wave with TE_1_ mode. **(d,e)** are the corresponding magnetic field patterns for the cases of inserting the dielectric material and PEC into the middle of GIMs, respectively. **(f)** is the magnetic field distribution from *x* = −200 *mm* to *x* = 200 *mm* (y = 0 *mm*) in (**e**). (**d**), (**e**,**f**) are the cases for the incident wave with TM_0_ mode.

In this section, we will show that the transmission effect in the straight waveguide with GIMs could be manipulated by inserting a defect inside the GIMs. First, we will give out the configuration of GIMs. The length of GIMs in region 1 and region 2 is 100 *mm*, *i.e.,* 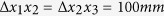. For the material parameters of GIMs given in Eq.(1) and (2), we set the gradient ratio 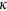 as 0.13/*mm*. Notice that this ratio could be arbitrarily chosen, the smaller this value is, the better the functionality is, hence as a price, the longer the gradient structure is. For a large value of 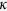, the reflection will be increased, hence with a worse functionality but with a shorter structure. For this case, the effective permittivity of GIMs in region 1 varies from 1 to 14, and it varies from 14 to 1 in region 2. A dielectric defect with effective permittivity 14 (its width is 6 *mm*) is inserted in the middle of region1 and region 2, to make the impendence matching at the interfaces between the defect and GIMs. When the wave with TE_1_ mode is incident from left port to right, obviously, we can find that the incident wave can be gradually converted into surface wave propagating in the GIMs as shown in Fig. 2(a). Then the surface wave in region 1 can pass through the dielectric defect marked by the black dashed frame. When the surface wave propagates in region 2, with the refractive index decreasing, the surface wave will be gradually converted into propagating wave. In such a propagating process, thanks to impendence matching, it almost is a total transmission. However, if the dielectric defect is replaced by a perfect electrical conductor (PEC), the surface wave in region 1 will be totally reflected back when it meets with the PEC defect. Hence, the total reflection could be achieved as shown in Fig. 2(b). To visualize the total reflection, we plot the electric field distribution from *x* = −200 *mm* to *x* = 200 *mm* (at y = 0 *mm*) in Fig. 2(b), and display it in Fig. 2(c), where we can find that the electric field in the right side is almost zero, which implies that a total reflection occurs. When the wave with TM_0_ mode is incident from left port to right, total transmission and reflection are realized by inserting the dielectric material and PEC defects, which are shown in Fig. 2(d, e) respectively. Likewise, from the magnetic field distribution from *x* = −200 *mm* to *x* = 200 *mm* (at y = 0 *mm*) in Fig. 2(e), which is plotted in Fig. 2(f), it is obvious that the total reflection happens in the waveguide with GIMs. Based on the above discussion, it is possible for us to manipulate the transmission effect in the waveguide, without considering the limitations of polarizations. It is noted that, by introducing ZIMs with defects^13^,^14^,^15^ into a waveguide, tunable transmission effect also could be realized. Such tunable transmission effect depends on the resonances^16^ of cavity modes, which is very sensitive to the material parameters of defects. Moreover, if tiny material loss of ZIMs is taken into consideration, transmission will be lowered. Hence, the method using ZIMs is not that feasible for practical application and our current design has more advantages over those using ZIMs.

In addition, if the GIMs is not placed exactly at the center of the waveguide, our structure still can effectively realize the functionality of mode conversion. To demonstrate this point, we carry out numerical simulations for the case of the GIMs shifting with a small distance from the center of the waveguide as shown in Supplementary Figure 2. From the simulated field patterns for TM_0_ incident wave in Supplementary Figure 2(a,b) corresponding to the shifting distance of 1 *mm* and 3 *mm* respectively, the mode conversion can still have a good performance, while the shifting distance will affect the outgoing propagating wave a little bit. When the shifting distance is tiny (*e.g.,* 1 *mm*), the wavefront of the outgoing propagating wave can be preserved well. However, when the shifting distance is large (*e.g.,* 3 *mm*), as the band structure of TM modes will be affected severely, other modes could also be excited, which will warp the outgoing wavefront. For the TE_1_ incident wave, by observing the field patterns in Supplementary Figure 2(c,d) related to the shifting distance of 1 *mm* and 3 *mm*, respectively, we find that the wave can almost perfectly experience a process of mode conversion with a well preserved wavefront. That’s because the shifting distance almost does not affect the band structure of TE modes, and the outgoing wavefront can be preserved well. Therefore, if the shifting distance is acceptable (*e.g.,* within 1 *mm*), the mode conversion in our waveguide structure with GIMs also have a commendable performance for both TE_1_ and TM_0_ modes, which means that our structure exhibits a good robustness.

### Waveguide bending effect

**Figure 3:**
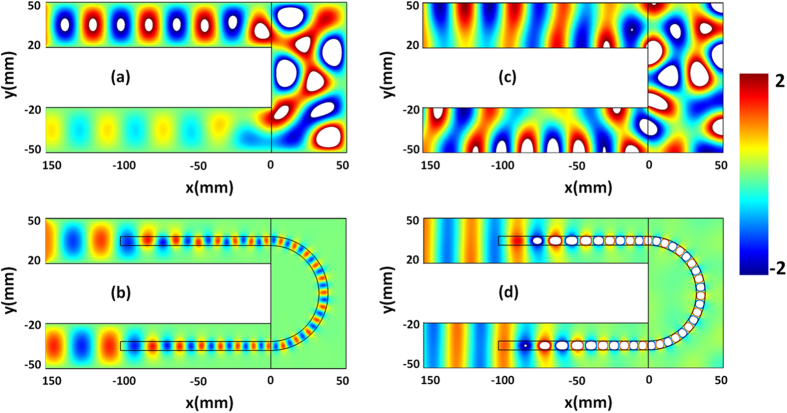
The simulated filed patterns for a waveguide bending structure. (**a**) is the electric field pattern in the bending waveguide without GIMs. **(b)** is the electric field pattern in the bending waveguide with GIMs. (**a**,**b**) are the cases for the incident wave with TE_1_ mode. **(c)** is the magnetic field pattern in the bending waveguide without GIMs. **(d)** is the magnetic field pattern in the bending waveguide with GIMs. (**c**,**d**) are the cases for the incident wave with TM_0_ mode.

As we have demonstrated that a straight waveguide with GIMs in Fig. 2 (a,d) can realize such a functionality: the propagating wave can be converted into surface wave and then the surface wave can also be converted into propagating wave. In particular, when it is at the region of GIMs with high refractive index, the surface wave will be tightly confined in the layer of GIMs. If we bend the region of waveguide with dielectric defect in Fig. 2 (a,d), it may not destroy the surface wave propagating along the GIMs, thus it is possible to realize bending waveguide. To verify this assumption, we propose a very common bending waveguide, as shown in Fig. 3(a). If the TE_1_ mode is incident from the upper port to the right side, when it runs into the bending part, strong reflection will appear, which brings about a low transmittance in the bottom port. However, by adding the GIMs into the waveguide, the transmission effect could be vastly improved with an intact wavefront as shown in Fig. 3(b). From the field pattern, we find that when the TE_1_ mode is incident from the upper port, the wave is gradually coupled into the GIMs and propagates as surface wave in GIMs. Particularly, the surface wave can perfectly pass through the curving part and gradually be converted into propagating wave. For the GIMs in Fig. 3(b), we divide it into three parts, where the upper and bottom straight parts are GIMs with effective permittivity varying from 1 to 14 (from *x* = −100 *mm* to *x* = 0 *mm*), and the curving part is composed of dielectric material with a constant effective permittivity 14. If the incident wave is TM_0_ mode, for the bending waveguide without GIMs as shown in Fig. 3(c), the incident wave is reflected and the wavefront is distorted in the bending waveguide. But with help of such GIMs, the incident wave with TM_0_ mode can be converted into surface wave, and passes through the curving part without reflection. Finally, with the refractive index of GIMs decreasing in the propagating process (from *x* = 0 *mm* to *x* = −100 *mm*), the surface wave will be gradually converted into propagating wave (see Fig. 3(d)). On basis of Fig. 3(b,d), almost perfect bending waveguide can be realized with the help of GIMs, which shows good performance for both TE_1_ and TM_0_ modes. For realizing perfect bending waveguide, transformation optics (TO) is a very good choice. However, in these designs^26^,^27^,^28^ based on TO, the required materials in the waveguide bends are anisotropic and inhomogeneous, thus very difficult for implementation. In contrast with the method of TO, our design can be realized by the isotropic dielectric materials, which is much easier to implement. In addition, zero index metamaterials (ZIMs) is another choice to realize bending waveguide effect. For example, by using anisotropic ZIMs without loss, almost perfect bending waveguide^11^ could be achieved theoretically. However, due to the anisotropic material property and the influence of loss, good bending waveguide effect^12^ will compromise in experiment.

### Waveguide splitting effect

**Figure 4:**
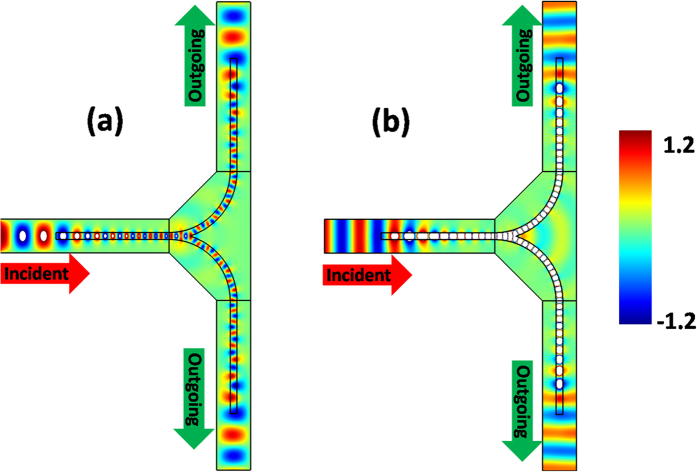
The simulated filed patterns for a waveguide splitting structure. (**a**,**b**) are the corresponding field patterns in a waveguide splitting structure with GIMs for TE_1_ and TM_0_ modes, respectively.

Based on the above bending waveguide structure with GIMs, we can also design a waveguide splitting structure. The key point of such a design is that it needs to divide the surface wave into more parts, and convert the parted surface waves into propagating waves. For the sake of simplicity, we divide the surface wave into two parts, and design the corresponding waveguide splitting structure as shown in Fig. 4. In the waveguide junction, the splitting structure is made of dielectric material with a constant effective permittivity 14. In the three straight waveguides with a width of 30 *mm*, the effective permittivity of GIMs varies from 1 to 14, and the width and length of GIMs are 6 *mm* and 100 *mm*, respectively. When the TE_1_ mode is incident from left port, it will be gradually converted into surface wave with the refractive index of GIMs increasing. By means of the splitting structure in the waveguide junction, the surface wave will be divided into two parts and then gradually transferred into two propagating waves with the decrease of the refractive index of GIMs (see Fig. 4(a)). Likewise, for the incident wave with TM_0_ mode, it follows similar propagating process with TE_1_ one, as shown in Fig. 4(b). By observing Fig. 4, such a splitting structure effectively divides the surface wave into two parts, and finally the parted surface waves can be converted into propagating waves, which commendably realizes the splitting effect of wave in a waveguide. We note that TO (such as conformal transformations) is also helpful in designing similar devices^29^, but our structure here is more practical and feasible yet with a little amount of scattering at the juncture due to the impedance mismatching.

### Broadband functionalities

**Figure 5:**
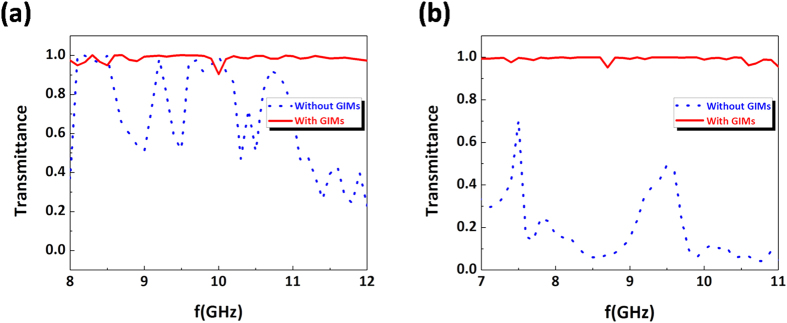
The transmittance *vs* frequencies for the case of the proposed bending waveguide in Fig. 3. (a,b) are the corresponding results for the incident wave with TE_1_ and TM_0_modes, respectively. In plots, the red solid curve is the case of waveguide with GIMs, while for the blue dot curve, it is the case of waveguide without GIMs.

For the above three designs in waveguides, we keep the working frequency as 9.5 GHz for both TE_1_ and TM_0_ modes. In fact, these designs can work for a broadband of frequencies, only if it can meet the condition, *i.e.,* the incident wave can be gradually converted into surface wave with the refractive index of GIMs increasing. Here we take the bending waveguide in Fig. 3 as an example to illustrate such a broadband feature. For TE_1_ mode in the waveguide with GIMs, when the frequency varies from 8 GHz to12 GHz, the transmittance will maintain around 100% (see red solid curve in Fig. 5(a)), which means that an almost total transmission has been achieved. While for waveguide without GIMs, the transmittance will be reduced at most frequencies by observing the blue dotted curve in Fig. 5(a). For the case of TM_0_ mode, when the waveguide is with GIMs, almost total transmission could be obtained in a broadband of frequencies, *i.e.,* from 7 GHz to 11 GHz (see red solid curve in Fig. 5(b)). When the waveguide is without GIMs, the transmittance will be reduced in the whole frequency range, which is shown by the blue dotted curve in Fig. 5(b). Therefore, our designs are not only independent of polarization, but also can work for a broadband of frequencies.

## Discussion

In conclusion, based on the concept of mode conversion, we have designed several meaningful devices of waveguides with GIMs. As the GIMs are comprised of isotropic dielectric materials, it is much easier to implement them in practical applications. More importantly, our proposed devices can work for a broadband of frequencies and are independent of polarizations. Recently, we have implemented a cloaking waveguide structure using similar technique^30^. We are expecting that, based on this new method of manipulating waveguide modes, it could bring about some new applications (*e.g.,* on-chip routing of light), and more experiments on this topic could be performed.

## Additional Information

**How to cite this article**: Fu, Y. *et al.* Applications of gradient index metamaterials in waveguides. *Sci. Rep.* **5**, 18223; doi: 10.1038/srep18223 (2015).

## Supplementary Material

Supplementary Information
