# Supplementary material for: Applications of gradient index metamaterials in waveguides
Source: Sci Rep. 2015 Dec 14;5:18223. doi: 10.1038/srep18223 (PMC4677397; doi:10.1038/srep18223)

**Supplementary Information for:**

**Applications of gradient index metamaterials in waveguides**

Yangyang Fu ^1^, Yadong Xu ^1, 2^ & Huanyang Chen ^2^

*College of Physics, Optoelectronics and Energy & Collaborative Innovation Center of Suzhou Nano Science and Technology, Soochow University,*

*No.1 Shizi Street, Suzhou 215006, China.*

***^1^*** *These authors contributed equally to this work*

***^2^*** [*ydxu@suda.edu.cn*](mailto:ydxu@suda.edu.cn) or [*chy@suda.edu.cn*](mailto:chy@suda.edu.cn)

**Figure S1 |** **The corresponding results for a high-index dielectric strip with a gradient thickness in a straight waveguide.** **(a)** The schematic diagram of a straight waveguide structure with a high-index dielectric strip with a gradient thickness, where region 1 (red region) is high-index dielectric strip with a gradient thickness (*i.e.,* zero thickness at the $x_{1}$ and $x_{3}$, and a maximal thickness *w* at $x_{2}$). **(b)** and **(c)** are the corresponding real part of electric and magnetic field patterns for the cases of the TE_1_ and TM_0_ incident wave, respectively, where the working frequency is 9.5 GHz, the maximal thickness of dielectric strip (its dielectric constant is 14) is 6*mm*, and $\Delta x_{1}x_{2}=\Delta x_{2}x_{3}=150mm$.


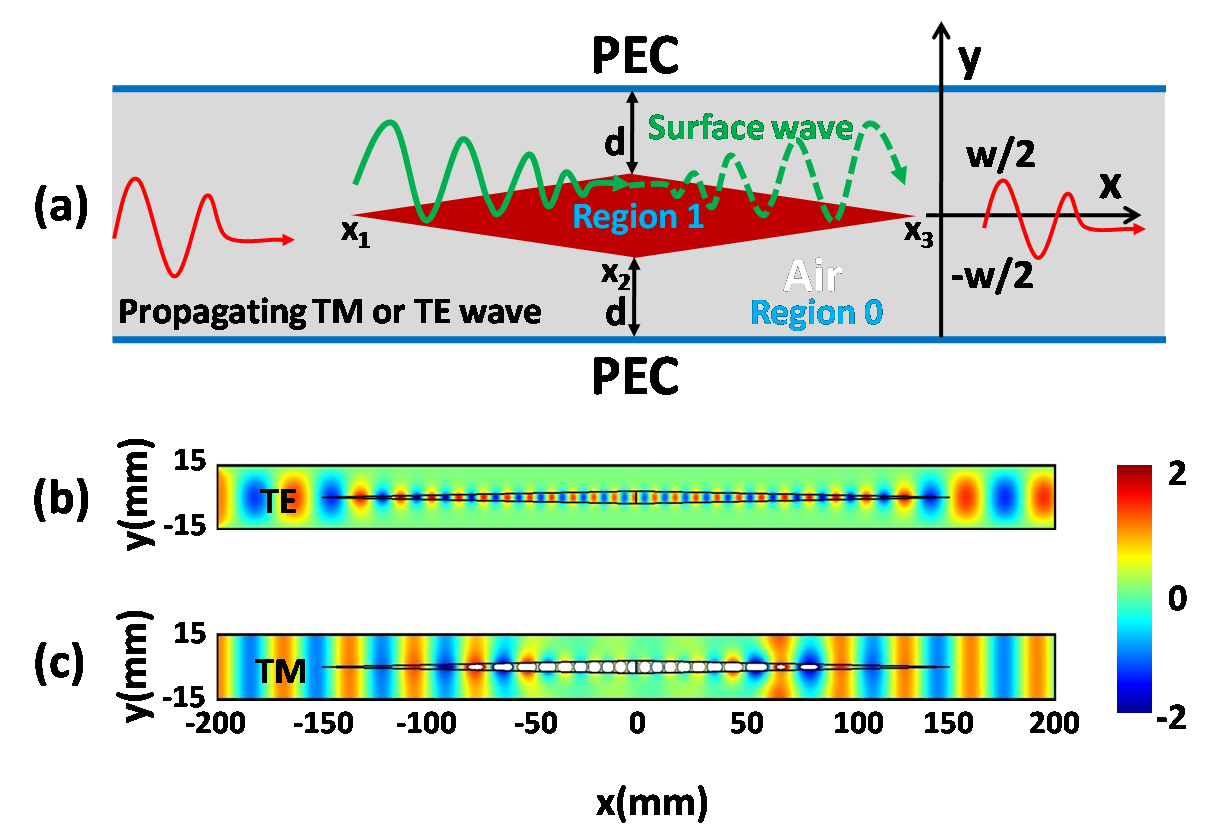


**Figure S2** | **The simulated results for the GIMs shifting with a little distance from the center of waveguide. (a)** and **(b)** are the corresponding real part of magnetic field for the cases of the GIMs shifting with a distance 1 *mm* and 3 *mm* from the center of waveguide, respectively, where the incident wave is TM_0_ mode. **(c)** and **(d)** are the corresponding real part of electric field for the cases of the GIMs shifting with a distance 1 *mm* and 3 *mm* from the center of waveguide, respectively, where the incident wave is TE_1_ mode. For the waveguide structure, it is same with one in the Fig.2(a, d) of the main text.


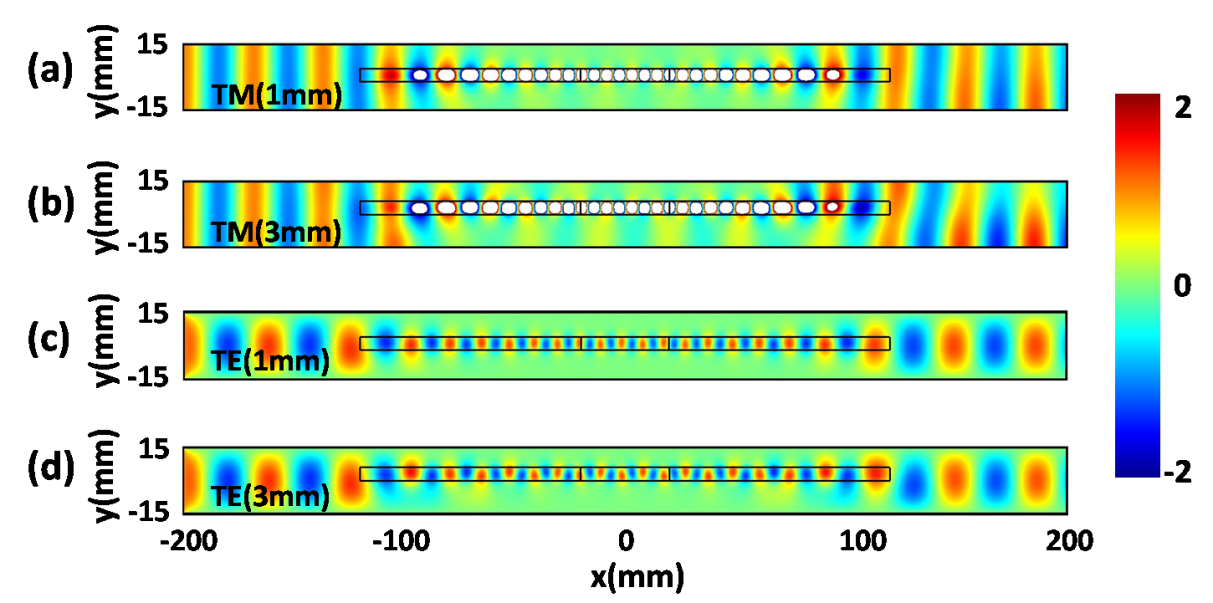

Supplement: Supplementary Information [file srep18223-s1.docx]
